# Impact of Water Content and Temperature on the Degradation of Cry1Ac Protein in Leaves and Buds of Bt Cotton in the Soil

**DOI:** 10.1371/journal.pone.0115240

**Published:** 2015-01-05

**Authors:** Mei-jun Zhang, Mei-chen Feng, Lu-jie Xiao, Xiao-yan Song, Wu-de Yang, Guang-wei Ding

**Affiliations:** 1 College of Agriculture, Shanxi Agricultural University, Taigu, People's Republic of China; 2 Department of Chemistry, Northern State University, Aberdeen, South Dakota, United States of America; USDA-ARS, United States of America

## Abstract

Determining the influence of soil environmental factors on degradation of Cry1Ac protein from Bt cotton residues is vital for assessing the ecological risks of this commercialized transgenic crop. In this study, the degradation of Cry1Ac protein in leaves and in buds of Bt cotton in soil was evaluated under different soil water content and temperature settings in the laboratory. An exponential model and a shift-log model were used to fit the degradation dynamics of Cry1Ac protein and estimate the DT_50_ and DT_90_ values. The results showed that Cry1Ac protein in the leaves and buds underwent rapid degradation in the early stage (before day 48), followed by a slow decline in the later stage under different soil water content and temperature. Cry1Ac protein degraded the most rapidly in the early stage at 35°C with 70% soil water holding capacity. The DT_50_ values were 12.29 d and 10.17 d and the DT_90_ values were 41.06 d and 33.96 d in the leaves and buds, respectively. Our findings indicated that the soil temperature was a major factor influencing the degradation of Cry1Ac protein from Bt cotton residues. Additionally, the relative higher temperature (25°C and 35°C) was found to be more conducive to degradation of Cry1Ac protein in the soil and the greater water content (100%WHC) retarded the process. These findings suggested that under appropriate soil temperature and water content, Cry1Ac protein from Bt cotton residues will not persist and accumulate in soil.

## Introduction

The cultivation of *Bacillus thuringiensis* crops has raised public concerns on their risk to nontarget organisms. Cry protein expressed exogenously in transgenic Bt (*Bacillus thuringiensis*) crops can enter into the soil ecosystem after secretion by crop roots [1]–[6], crop aboveground and post-harvest residues returning to soil [7]–[9], and pollen dissemination [10]. Once it enters the soil, Cry protein is rapidly absorbed and bound on surface-active particles, including clay minerals, humic acids, and complexes of montmorillonite-humic acids-Al hydroxypolymers [11]–[17]. The binding of Cry protein reduces its availability to microbes, which is likely responsible for the persistence of Cry protein in soil [7], [13], [14], [18]. Thus, degradation dynamic characteristics and amount of Cry protein residues in soil are a core issue for assessing the ecological risk of transgenic Bt crops in soil.

The previous researchers have carried out the analysis of the degradation of purified Cry protein and Cry protein released by transgenic Bt crops in soil [19], [20]. The degradation rate has been found to be varying significantly, probably due to the diversity of materials, soil type, and experimental methods used. Donegan et al. [21] reported that the purified protein from *Bacillus thuringiensis* subsp. *kurstaki* and the Cry1Ac protein from Bt cotton persisted at detectable levels as measured by enzyme-linked immunosorbent assay (ELISA) for up to 28 and 56 d in soil, respectively. Sims and co-workers [22], [23] performed a relevant insect bioassay and reported that the DT_50_ (50% degradation time) and DT_90_ (90% degradation time) of degradation of Cry1Ab protein released from Bt corn tissue in soil were 1.6 d and 15 d, respectively. The DT_50_ of Cry2A protein in Bt cotton were 15.5 d and 31.7 d in laboratory and field degradation, respectively. The Cry1Ab toxin was detected in soil for 180 d from root exudates after growth of Bt corn [3] and from biomass of Bt corn 3 years after incorporation into soil [24], the longest period evaluated in both cases. The DT_50_ and DT_90_ of degradation of Cry1F protein isolated from a recombinant *Pseudomonas fluorescens* strain in soil were 0.6 d and 6.9 d, respectively [25]. Zwahlen et al. [8] detected Cry1Ab protein after 200–240 d of Bt corn tissues being buried into the soil. A study by Bai et al. [26] demonstrated that, in a marine-fluvigenic yellow loamy paddy soil, pale paddy soil on quaternary red soil, and blue clayey paddy soil, the degradation of Cry1Ab protein in KMD1 stems and leaf blades was more rapid during the early period (12 d after treatment). However, the degradation rate varied, with the DT_50_ value of degradation being 2.2–11.6 d. The Cry1Ab protein in Bt corn showed the fast negative exponential degradation in soil in the early period. On the contrary, in the later period, Cry1Ab protein degradation was relative stable and slow. The DT_50_ and DT_90_ of degradation of Cry1Ab protein were 6.8 d and 21.76 d, and 0.54 d and 7.12 d, respectively when straws were buried in soil or covered on soil surface [27]. Furthermore, other factors (such as soil properties, microbes, water content, pH, and temperature) were also found to have impact on the degradation of Cry protein in soil [19], [20], [28]–[31].

Obviously, the previous investigations on the degradation dynamics of Cry protein in transgenic Bt crops were mostly concerned with Bt corn and rice straws. However, according to the report by the International Service for the Acquisition of Agri-biotech Applications (ISAAA), the global planting area of transgenic cotton reached 24.7 million ha in 2011, accounting for 68.6% of the total cotton plantation. The insect-resistant transgenic Bt cotton is a major cotton variety used around the world. With the Bt cotton being planted in China in 1997, the planting area of Bt cotton has been expanding rapidly. As an example, in 2011 the planting area of Bt cotton reached 3.9 million ha in China, accounting for 71.5% of the national total cotton plantation [32]. The dropping of flower, buds, and boll of cotton is a widespread phenomenon in production, with the typical dropping rate of 60–70% or even 80% in serious cases [33]. In addition, the different cultivation techniques such as thinning of seedlings, final singling, topping, and pruning during cotton growing period are implemented. Aboveground residues resulting from these procedures as well as residual stubbles are returned to the field by tillage. The soil water content and temperature are variable factors with the climates. Hence, increasing attention has been paid to the effects of soil water content and temperature on the Cry1Ab protein released from Bt corn and rice straw [28], [31]. To date, however, there is a limited study about the degradation dynamics of Cry1Ac protein in Bt cotton residues under different conditions of soil water content and temperature.

This study aims to determine the relationship of Cry1Ac protein degradation with soil water content and temperature. In this study, transgenic Bt cotton (expressing the Cry1Ac protein) was selected as the study target. The degradation of Cry1Ac protein in leaves at the thinning stage and in buds in soil under different soil water content and temperature were carried out by ELISA. The exponential model and a shift-log model were initiated to fit the degradation dynamics of Cry1Ac protein in soil, and DT_50_ (50% degradation time) and DT_90_ (90% degradation time) values were calculated. The analysis of variance (ANOVA) was implemented to identify the different impacts of soil temperature and moisture on Cry1Ac degradation rate. The results of the present work are expected to be useful for assessing the ecological risks of Bt cotton planting.

## Materials and Methods

### Soil

The trial soil was collected from a 0 to 15 cm layer at an agricultural experimental cotton field in Shanxi Agriculture University (China). The experimental field has never been planted with transgenic Bt crops before our study. The soil sample was air-dried at room temperature and passed through a 2 mm sieve, and all large stones and plant debris were removed by hand. The soil was classified as calcareous drab soil (semiarid Alfisols). The soil was a land loam. The soil had a pH of 8.32 (H_2_O); 56% sand; 36.70% silt; 7.30% clay; 13.80 g kg^−1^ organic matter; 0.93 g kg^−1^ total nitrogen; 53.24 mg kg^−1^ alkaline hydrolyzed nitrogen; 7.46 mg kg^−1^ available phosphorus; and 187.63 mg kg^−1^ available potassium. The soil is suitable for the cotton planting. To data, there is no study concerning with the degradation of the Cry1Ac protein in such soil (Shanxi). We need to note that the field study was authorized by Shanxi Agricultural University (P. R. China). In addition, no specific permissions were required for these locations/activities because the research activities were for the local agricultural service. Furthermore, the field studies did not involve endangered or protected species and this study also did not involve vertebrate species.

### Bt cotton

The transgenic Bt cotton variety used in the study was Jinmian 26 (expressing the Cry1Ac protein), which was derived from a commercial cotton variety Jinmian 7 and transformed by *Agrobacterium* infection. The seeds were provided by the Cotton Research Institute, Shanxi Academy of Agricultural Sciences (China).

The seeds of Jinmian 26 were planted on the experimental farm of Shanxi Agricultural University with row spacing of 75 cm and seedling distance of 15 cm. Basal carbamide of 240 kg ha^−1^, diammonium phosphate of 450 kg ha^−1^, and potassium sulfate of 225 kg ha^−1^ were applied before sowing. With an appropriate preparation for germination, the seeds were sowed by bunch planting (four seeds in each planting hole) with plastic film mulching on April 28, 2012. The seedlings were thinned at the trefoil stage. No pesticide was used and other typical management procedures were implemented. Leaves at the thinning stage and young buds at 3 d on the first to the third branches at budding stage were collected. A proportion of the samples was cut into short strips and crushed in liquid nitrogen for Cry1Ac protein determination. The Cry1Ac protein content was found to be 5794.68 ng g^−1^ (standard deviation = 121.38; n = 3) (dry weight) in the leaves and 2386.81 ng g^−1^(standard deviation = 86.47; n = 3) (dry weight) in the buds by ELISA analysis. Other parts of the samples were treated with liquid nitrogen and made into lyophilized powder for testing degradation of Cry1Ac protein under different combinations of soil water content and temperature.

### Experimental design

Our experimental design consisted of two soil factors, namely soil water content and temperature. Three levels of soil water contents (50%, 70%, and 100% water-holding capacity (WHC)) and three levels of soil temperatures (15°C, 25°C, and 35°C) were implemented. A two-factor completely randomized design was adopted. Nine treatments were initiated for each of the leaf and bud samples. Each treatment was prepared with 18 replicates of which three replicates were destructively-sampled at each of the six time intervals.

Soil water-holding capacities of 50%, 70%, and 100% were maintained as the follows. A total of 0.40 g of lyophilized powder of leaves or buds and 3.00 g of soil were mixed evenly and placed into a 10 mL centrifuge tube. Distilled water in the amount of 0.38 g, 0.55 g, and 0.77 g was added to each tube to achieve water contents of 12.62%, 18.49%, and 25.67%, respectively. The samples were weighed every 4 d and water was added to maintain constant water content of soil in the tube. Three small holes (each with diameter of 2 mm) were made in the tubes for air exchange. The Cry1Ac protein contents in the soil from the initial stage of degradation under 50%, 70%, and 100% of soil water holding capacity were determined as 613.19, 586.8, and 555.84 ng g^−1^ soil, respectively for the leaves; and 252.57, 241.70, and 228.95 ng g^−1^ soil, respectively for the buds.

The tubes were placed in the thermostat heating blocks set at 15°C, 25°C, and 35°C under humidity of 70% and incubated in a dark condition.

The samples were collected on 16, 32, 48, 64, 80, and 96 d. For each treatment, three tubes (triplicates) were taken out and stored at −80°C for analyzing Cry1Ac protein content by ELISA.

### The Determination of Cry1Ac protein content

The Bt protein content was measured using a commercial ELISA kit (Center of Crop Chemical Control, China Agricultural University, Beijing) according to the protocol described by Wang et al. [34]. Briefly, two steps were used in a preparation for Cry1Ac protein: extraction and quantification. To extract Bt (Cry1Ac) protein from the soil samples, 1.00 g leaf-soil or bud-soil mixture sample was mixed with a buffer solution consisting of 1.33 g sodium carbonate (Na_2_CO_3_), 0.19 g Dithiothreitol (DTT), 1.46 g sodium chloride (NaCl), 0.50 g vitamin C, and 250 mL distilled water and then homogenized. Immediately after homogenization, the soil–buffer mixture was centrifuged at 6000 r/min for 10 min. After centrifugation, an aliquot was removed from the supernatant to analyze. For quantification of Cry1Ac protein in test samples, the well of microtiter plate was first coated with a primary monoclonal anti-Cry1Ac antibody. A secondary polyclonal anti-Cry1Ac antibody with enzyme and the test sample were then added to the plate. The plate was incubated for 30 min at 37°C. After the incubation, the plate was washed using the phosphate buffer solution and then treated with substrate buffer solution with O-phenylenediamine (OPD) that acted with the enzyme. After subsequent incubation for 15–20 min at 37°C, the 50 µL of 2 mol L^−1^ sulfuric acid (H_2_SO_4_) was added to the well to end the action, and the mixture was analyzed by a Multiskan Ascent spectrometer at 492 nm (Thermo Co., Finland). A six-point standard curve of purified Cry1Ac (supplied with the kit) was used for analysis and comparison.

### Degradation model

The expression for exponential model (the first-order) was Y (t)  =  Ae^−kt^. Y (t) was the amount of residues of Cry1Ac protein in soil at time t, A was the amount of Cry1Ac protein in soil at the initial time, k was the Cry1Ac protein degradation rate constant, t was the degradation time (d). The expressions for shift-log model was Y (t)  = B×(t+k)^m^. Y(t) and t were the same as that of the exponential model. B, k, and m were the Cry1Ac protein degradation rate constant. The DT_50_ (50% degradation time) and DT_90_ (90% degradation time) values under different settings of soil water content and temperature were estimated by using the model in Excel (Microsoft, USA).

### Statistical analysis

The statistical analysis of the amount of residue and degradation rate of Cry1Ac protein at each sampling time in the soil as well as the fitting of degradation equation was carried out using Excel and DPS (data processing system) software. A level of P<0.05 was considered as statistically significant. The analysis of variance (ANOVA, two-way) statistical method was implemented to compare the different impacts of soil temperature and moisture on the Cry1Ac degradation rate (P<0.01).

## Results

### The degradation dynamics of Cry1Ac protein in soil under the same water content and different temperatures

**Figure 1:**
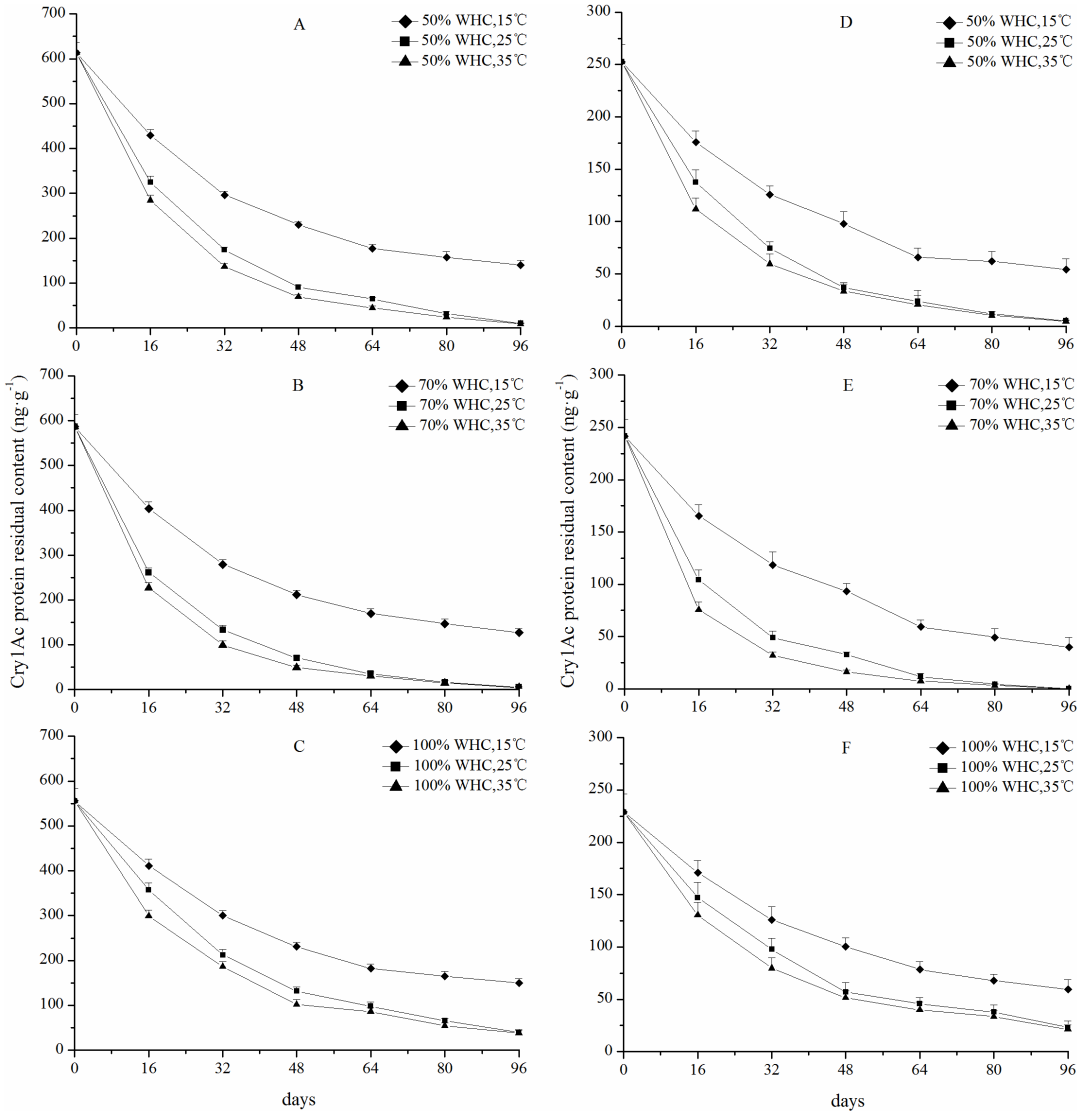
The degradation dynamics of Cry1Ab protein in leaves (A, B, C) and buds (D, E, F) with the soil same water content and different temperatures settings. The data are expressed as the mean of three replicates ± standard deviation).

The degradation dynamics of Cry1Ac protein in leaves and buds in soil under the same water content and different temperatures are shown in Fig. 1 (A, B, C, D, E and F). Generally speaking, Cry1Ac protein degradation exhibited the same dynamic trend under the same soil water content at different temperatures during the sampling period. For example, on day 48, 56.18–93.26% of the initial amount of Cry1Ac protein had been degraded under all treatment conditions. After day 48 for each treatment, no significant difference was observed in the percentage of residual Cry1Ac protein compared to adjacent sampling periods, indicating that Cry1Ac degradation had been gradually slowed down.

The content of Cry1Ac protein remaining in the leaf-soil mixture differed significantly (P<0.05) among the different incubation temperatures with a single water holding capacity before day 48 (Fig. 1. A, B, C). A similar trend before day 48 was also observed in the bud-soil mixture at the 70% water holding capacity (Fig. 1. E). In addition, the amounts of residues of Cry1Ac protein in the bud-soil mixture were significantly (P<0.05) different at different temperatures for the groups with 50% and 100% water holding capacities before day 32 (Fig. 1. D, F). The amounts of residues of Cry1Ac protein in the leaf-soil and bud-soil mixture at 35°C was significantly lower (P<0.05) than at 25°C and 15°C with a single water holding capacity before day 32 (Fig. 1. A, B, C, D, E and F). In the later stage (days from 64 to 96), the amount of residues of Cry1Ac protein in leave-soil and bud-soil mixture was not significantly different at both 35°C and 25°C. However, both were significantly lower (P<0.05) than that at 15°C (Fig. 1. A, B, C, D, E and F). On day 96, in the end of sampling period, the differences in the amount of residues of Cry1Ac protein in leaf-soil mixture among the different incubation temperatures with a single water holding capacity could still be detected. For example, under 70% water holding capacity and soil temperature of 15°C, the amount of residues of Cry1Ac protein was 127.32 ng g^−1^ (standard deviation = 10.47; n = 3), which was 28.55 and 23.32 times as that of at 35°C and 25°C, respectively (Fig. 1. B). Cry1Ac protein residues could be detected in bud-soil mixture at both 50% and 100% water holding capacity under different temperatures (Fig. 1. D, F). Although 16.46% of the initial protein amount could be still detected at 15°C, there was no Cry1Ac protein observed in bud-soil mixture at both 35°C and 25°C with 70% water holding capacity (Fig. 1. E). Based on these findings, our results indicated that higher temperature facilitates the degradation of Cry1Ac protein in the soil. And these observations suggested that at appropriate soil temperature and water content, Cry1Ac protein will not persist and accumulate in soil.

### The effect of soil water content and temperature on degradation rate of Cry1Ac protein

As shown in Table 1 and Table 2, there were significant effects of soil temperature and water holding capacity on the degradation rates of Cry1Ac protein in leave-soil and bud-soil mixture. The significant effects of interaction of temperature and water holding capacity occurred before day 32 (P<0.01). Under a single temperature, the degradation rates of Cry1Ac protein in leave-soil and bud-soil mixture at 100% water holding capacity was significantly lower (P<0.05) than those at 50% and 70% water holding capacity throughout the entire sampling period. The degradation rates of Cry1Ac protein in leave-soil and bud-soil mixture with 70% soil water holding capacity and soil temperature of 25°C and 35°C before day 32 or day 48 were significantly higher (P<0.05) than those under 50% water holding capacity. These results illustrated that excessive soil water content (e.g., 100% WHC) would retard the degradation of Cry1Ac protein in the soil. On the other hand, moderate soil moisture content (e.g., 70% WHC) was conducive to a rapid degradation of Cry1Ac protein in the early stage. The degradation rates of Cry1Ac protein under 25°C and 35°C with different soil water contents were significantly higher (P<0.05) than those at 15°C with different soil water content over the entire sampling period. This demonstrated that temperature was a major factor affecting the degradation of Cry1Ac protein in the soil. Across all treatment conditions, the degradation rates of Cry1Ac protein in leave-soil and bud-soil mixture at 35°C with 70% water holding capacity were the highest before day 32 (Table 1) and day 48 (Table 2), respectively. The lowest degradation rate of Cry1Ac protein was observed at 15°C with 100% water holding capacity over the entire sampling period.

**Table 1:** The degradation rates (%) of Cry1Ac protein in leaves under different soil water content and temperature settings (data reported as the mean of three replicates ± standard deviation).

Temperature	Water holding capacity	Days of degradation (d)					
		16	32	48	64	80	96
15°C	50%	30.21±2.21e	51.64±1.84e	62.44±2.24e	71.23±2.05c	74.27±1.19c	77.11±1.12c
	70%	31.11±2.54e	52.34±2.16e	63.98±2.83e	71.10±2.44c	74.93±2.44c	78.03±1.08c
	100%	26.00±1.86f	46.04±2.03f	58.44±1.96f	67.23±3.16d	70.27±3.18d	73.11±1.45d
25°C	50%	47.06±2.30c	71.52±3.17c	85.28±2.43bc	90.00±2.87a	94.87±2.06a	98.33±1.98a
	70%	55.45±1.75b	77.25±2.94b	88.02±3.18ab	94.00±1.99a	97.23±1.98a	99.07±2.01a
	100%	35.79±2.37d	61.76±3.46d	76.34±2.66d	82.31±2.00b	88.19±2.11b	92.77±2.87b
35°C	50%	53.57±1.83b	77.71±3.87b	88.67±3.44ab	92.78±2.65a	96.12±3.21a	98.59±3.44a
	70%	61.28±1.94a	83.14±4.01a	91.56±2.67a	94.79±2.35a	97.52±2.66a	99.24±2.56a
	100%	46.23±3.04c	66.63±3.26cd	81.70±1.11c	84.52±1.87b	90.11±1.88b	93.18±2.11b
Two- way ANOVA(F value)							
Temperatures		572.55**	426.24**	522.47**	176.56**	227.40**	265.27**
Water holding capacity		159.17**	64.89**	60.18**	40.94**	21.36**	18.55**
Interaction(T×WHC)		16.90**	4.94**	2.55	2.54	0.66	0.12

Different letters in the same column indicate statistically significant difference (p<0.05). ** indicate that the degradation percent of Bt protein is significantly influenced by soil water content, temperature and their interaction (p<0.01).

**Table 2:** The degradation rates (%) of Cry1Ac protein in buds under different soil water content and temperature settings (data reported as the mean of three replicates ± standard deviation).

Temperature	Water holding capacity	Days of degradation (d)					
		16	32	48	64	80	96
15°C	50%	30.43±4.23e	50.18±2.55e	61.19±3.59d	73.86±4.23d	75.49±3.05d	78.62±4.11c
	70%	31.46±3.27e	51.01±3.43e	62.46±2.89d	75.49±3.65d	79.66±2.74cd	83.54±3.87c
	100%	25.33±1.84f	44.89±2.89f	56.18±3.11e	65.69±2.98e	70.43±1.99e	73.89±2.98d
25°C	50%	45.53±2.18c	70.54±3.65c	85.39±2.56b	90.66±3.12b	95.27±2.87a	97.98±3.05a
	70%	56.88±3.01b	79.84±4.77b	86.45±4.44b	95.24±2.87ab	98.22±3.43a	100 a
	100%	35.67±1.99d	57.29±1.98d	75.18±3.87c	80.00±1.94c	83.49±1.09bc	89.97±3.18b
35°C	50%	55.65±2.45b	76.48±2.65b	86.77±4.01b	91.83±2.69ab	95.98±2.84a	98.12±2.99a
	70%	68.78±3.06a	86.87±3.49a	93.26±3.45a	96.88±3.18a	98.69±3.05a	100 a
	100%	43.00±1.08c	65.18±4.11c	77.48±2.91c	82.56±2.22c	85.37±2.97b	90.64±3.23b
Two- way ANOVA(F value)							
Temperatures		341.89**	165.21**	153.60**	97.67**	124.10**	103.01**
Water holding capacity		295.59**	58.16**	25.35**	41.25**	49.52**	25.27**
Interaction(T×WHC)		21.51**	5.77**	1.89	0.66	1.42	0.36

Different letters in the same column indicate statistically significant difference (p<0.05). ** indicate that the degradation percent of Bt protein is significantly influenced by soil water content, temperature and their interaction (p<0.01).

### Degradation model, DT_50_ and DT_90_ of Cry1Ac protein under different water content and temperature settings

On examining the data, the degradation characteristics of Cry1Ac protein in leave-soil and bud-soil mixture complied with the exponential model and the shift-log model under different soil water contents and temperatures (Table 3, 4). In all treatment conditions, the fitting precision of the shift-log model was less accurate under 50% water holding capacity and 35°C in bud-soil mixture, and under 70% water holding capacity and 35°C in leaf-soil mixture, with the r values of 0.7899 (p = 0.1435) and 0.4486 (p = 0.7336), respectively. However, the correlation coefficients of the exponential model for Cry1Ac protein degradation dynamics varied in the range of 0.9892 - 0.9998 (p<0.01) for all soil water content and temperature treatments. Based on this analysis, the exponential model was chosen to estimate the DT values of Cry1Ac protein degradation.

**Table 3:** Degradation model, DT_50_ and DT_90_ of Cry1Ac protein in leaves in the soil.

Condition	Degradation Model		r	p	DT_50_	DT_90_
50% WHC, 15°C	Exponential Model	Y = 592.08e^-0.0185t^	0.9892	<0.001	35.64	122.76
	Shift-log Model	Y = 3.30×10^5^(t+55.96)^−1.5610^	0.9986	<0.001	-	-
50% WHC, 25°C	Exponential Model	Y = 611.36e^−0.0389t^	0.9995	<0.001	17.75	59.15
	Shift-log Model	Y = 3.37×10^9^(t+74.08)^−3.6026^	0.9980	<0.001	-	-
50% WHC, 35°C	Exponential Model	Y = 610.34e^−0.0461t^	0.9995	<0.001	14.93	49.81
	Shift-log Model	Y = 2.14×10^9^(t+62.86)^−3.6372^	0.9991	<0.001	-	-
70% WHC, 15°C	Exponential Model	Y = 565.68e^−0.0189t^	0.9894	<0.001	34.65	119.61
	Shift-log Model	Y = 2.87×10^5^(t+53.79)^−1.5528^	0.9991	<0.001	-	-
70% WHC, 25°C	Exponential Model	Y = 582.81e^−0.0470t^	0.9993	<0.001	14.60	48.82
	Shift-log Model	Y = 8.37×10^9^(t+67.19)^−3.9144^	0.9992	<0.001	-	-
70% WHC, 35°C	Exponential Model	Y = 583.62e^−0.0560t^	0.9990	<0.001	12.29	41.06
	Shift-log Model	Y = −287.27(t−17.39)^−0.4113^	0.4486	0.7336	-	-
100% WHC, 15°C	Exponential Model	Y = 539.40e^−0.0161t^	0.9900	<0.001	41.07	140.75
	Shift-log Model	Y = 4.11×10^5^(t+66.68)^−1.5710^	0.9981	<0.001	-	-
100% WHC, 25°C	Exponential Model	Y = 555.31e^-0.0286t^	0.9981	<0.001	24.27	80.60
	Shift-log Model	Y = 1.00×10^9^(t+89.14)^−3.2049^	0.9976	<0.001	-	-
100% WHC, 35°C	Exponential Model	Y = 544.91e^−0.0332t^	0.9961	<0.001	20.29	68.80
	Shift-log Model	Y = 5.12×10^8^(t+74.81)^−3.1827^	0.9993	<0.001	-	-

**Table 4:** Degradation model, DT_50_ and DT_90_ of Cry1Ac protein in buds in the soil.

Condition	Degradation Model		r	p	DT_50_	DT_90_
50% WHC, 15°C	Exponential Model	Y = 245.55e^−0.0189t^	0.9855	<0.001	35.11	120.06
	Shift-log Model	Y = 9.13×10^4^(t+49.40)^-1.5091^	0.9985	<0.001	-	-
50% WHC, 25°C	Exponential Model	Y = 252.92e^−0.0385t^	0.9998	<0.001	18.05	59.87
	Shift-log Model	Y = 4.25×10^9^(t+79.90)^−3.7949^	0.9973	<0.001	-	-
50% WHC, 35°C	Exponential Model	Y = 249.58e^−0.0453t^	0.9981	<0.001	15.02	50.56
	Shift-log Model	Y = −556.83(t−1.1450)^−5.8374^	0.7899	0.1435	-	-
70% WHC, 15°C	Exponential Model	Y = 237.41e^−0.0205t^	0.9974	<0.001	32.91	111.35
	Shift-log Model	Y = 2.74×10^5^(t+52.47)^−1.7751^	0.9991	<0.001	-	-
70% WHC, 25°C	Exponential Model	Y = 239.97e^−0.0490t^	0.9983	<0.001	14.00	46.84
	Shift-log Model	Y = 2.53×10^9^(t+64.13)^−3.8838^	0.9988	<0.001	-	-
70% WHC, 35°C	Exponential Model	Y = 240.48e^−0.0676t^	0.9985	<0.001	10.17	33.96
	Shift-log Model	Y = 5.25×10^8^(t+45.23)^−3.8278^	0.9999	<0.001	-	-
100% WHC, 15°C	Exponential Model	Y = 223.12e^−0.0158t^	0.9940	<0.001	42.17	143.90
	Shift-log Model	Y = 1.07×10^6^(t+87.64)^−1.8887^	0.9993	<0.001	-	-
100% WHC, 25°C	Exponential Model	Y = 226.44e^−0.0259t^	0.9970	<0.001	26.35	88.54
	Shift-log Model	Y = 6.46×10^8^(t+104.61)^−3.1898^	0.9972	<0.001	-	-
100% WHC, 35°C	Exponential Model	Y = 222.92e^−0.0295t^	0.9934	<0.001	22.59	77.12
	Shift-log Model	Y = 1.31×10^8^(t+80.56)^−3.0198^	0.9987	<0.001	-	-

Under the same soil water content, the DT_50_ and DT_90_ values were the least at 35°C. For the same temperature, a DT_50_ and DT_90_ value was the least observed under 70% water holding capacity (Table 3 and 4). According to the data, among all treatment conditions, the largest degradation rate of Cry1Ac protein was observed at 70% soil water holding capacity and temperature of 35°C°C, with a DT_50_ and DT_90_ of 12.29 d and 41.06 d in leaf-soil mixture (Table 3), respectively; and 10.17 d and 33.96 d in bud-soil mixture (Table 4), respectively. Simultaneously, the least degradation rate of Cry1Ac protein was found at 100% soil water holding capacity and temperature of 15°C, with a DT_50_ and DT_90_ of 41.07 d and 140.75 d in leaf-soil mixture (Table 3), respectively; and 42.17 d and 143.90 d in bud-soil mixture (Table 4), respectively.

## Discussion

The soil samples examined in this study contained a mixture of the soil and leaves or buds. Cry1Ac protein initially from leaves or buds and the Cry1Ac protein released into the soil were examined by ELISA. Up to day 48, 56.18–93.26% of the initial amount of Cry1Ac protein in leaves or in buds had already been degraded under different water contents and temperatures. This indicated that Cry1Ac protein underwent rapid early degradation in the soil. Previous studies have shown that free Cry protein from *Bacillus thuringiensis* subsp. *kurstaki* or *tenebrionis* are readily utilized as a sole source of carbon and/or nitrogen by pure and mixed cultures of microbes in soil suspensions [12]. The quantity of Cry1Ab protein in the decomposing Bt corn and soil mixture declined rapidly during the incubation [35]. These phenomena probably account for the rapid degradation of Cry1Ac protein in leaf-soil and bud-soil mixture during the early phase in current study. Notably, the present result is consistent with the degradation of Cry proteins in Bt rice material-soil mixture [26], [28], Bt corn material-soil mixture [8], [27], [30], [31], [36], [37] and Bt cotton material-soil mixture [23], [38], [39], and a degradation of purified Cry1Aa protein in soil [20] in the early phase. As Cry1Ac protein cannot be absorbed into soil colloids until it has been released from the matrix of the residue, such rapid degradation during the early period strongly influences the persistence of Cry1Ac protein in the soil [35].

Active particles such as soil organic matter and/or humic substances possess a unique surface area and ion exchange capacity [40]. It has been found that the active particles in soil could absorb Cry proteins [11]–[17]. The bound Cry proteins were not utilized as a source of carbon, slightly as a source of nitrogen; and they did not support growth in the absence of an exogenous source of both available carbon and nitrogen [12]. Crecchio and Stotzky [13] showed that under laboratory conditions, Cry protein from *Bacillus thuringiensis* subsp. *kurstaki* bound to soil humic acids degraded more slowly than free protein. The Cry protein released from root exudates and upon disintegration of transgenic Bt crops into the soil would be in a free state susceptible to biodegradation only briefly [41]. Thus, inhibited microbial degradation of Cry protein adsorbed by soil particles and the persistence of Cry protein in relatively recalcitrant residues of Bt crops could be the major reason for a slow degradation of Cry1Ac protein in leaf-soil and bud-soil mixture in later periods. This was consistent with the slowed degradation of Cry protein of in Bt rice material-soil mixture [26], [28], Bt corn material-soil mixture [8], [27], [30], [31], [36], [37], Bt cotton material-soil mixture [23], [38], [39], and degradation of purified Cry1Aa protein [20] in later periods.

At the end of the sampling period, no Cry1Ac protein in bud-soil mixture was detected under 70% water holding capacity and temperatures of 35°C and 25°C. This result was similar to the report that no detectable Cry3Bb1 protein was found under laboratory conditions after 21 d in montmorillonite-amended soil and after 40 d in kaolinite-amended soil to which different amount of Bt corn residues expressing the Cry3Bb1 protein added [9]. The field studies on the persistence of Cry proteins released by transgenic Bt crops were generally in good agreement with laboratory studies. A field-accumulation study, which monitored a field where Bt cotton was cropped for 3 to 6 years in succession and residues were subsequently incorporated into the soil by post-harvest tillage, found no detectable soil residues of Cry1Ac protein as determined by ELISA and insect bioassays after the last season's tillage [42]. The low concentration of Cry3Bb1 detected in the rhizosphere soil and the fact that there was no increase in detectable Cry3Bb1 during 3 years of subsequent MON88017 (Bt corn) cultivation support the conclusion that there was no accumulation of this protein in the soil [6]. The amount of Cry1Ab/Cry1Ac protein in the rhizosphere soil of Bt rice was only significantly higher than that of the basal level during the first two months after harvest, indicating that the protein will not persist and accumulate in soil [43].The findings of the present study were in agreement with these results and demonstrated that Cry protein in the soil can be completely degraded in soil under an appropriate environmental condition.

One of the findings of this study was that temperature was a major factor controlling Cry1Ac protein degradation in the soil. For the same level of water holding capacity, higher temperature could facilitate degradation of Cry1Ac protein in the soil. It has been shown that an increase of 10°C in soil temperature can result in two- to three-fold increase in microbial activity [44]. Thus, the increase in activity of soil microbes at higher temperatures may explain the faster degradation of Cry1Ac protein we observed at higher temperatures. This result was consistent with those of research on Cry1Ab protein degradation in Bt rice and Bt corn [2], [8], [28], [31]. Feng et al. [31] investigated the degradation of Cry1Ab protein in the straw of Bt corn and did not find any significant effect of different soil water contents on Cry1Ab protein degradation. Bai et al. [26] found that Cry1Ac protein in the leaves of Bt rice was more likely to be degraded under waterlogged conditions (before 12 d); they further proposed that this may be due to the increase in quantity and activity of particular types of soil microbes by waterlogging. However, our result showed that the greater water holding capacity (e.g., 100%) was unfavorable for the degradation of Cry1Ac protein in leaf-soil and in bud-soil mixture; while moderate water holding capacity of soil (e.g., 70%) was more conducive to the rapid degradation of Cry1Ac in the early period. Thus, our results suggested that the quantity and activity of certain types of microbes in the soil may be larger and stronger at proper soil water content in dry farmlands.

Establishing the best fit model for the dynamics of degradation is important for deriving the proper DT_50_ and DT_90_ values. So far, numerous researchers have chosen a diversity of models for fitting Cry protein degradation dynamics. Sims and Holden [22] used the exponential model to fit the degradation of Cry1Ab protein in the straw of Bt corn in the soil. They further reported that the DT_50_ and DT_90_ values were fitted as 1.6 d and 15 d, respectively. Herman et al. [25] employed the biexponential model to estimate the DT_50_ and DT_90_ values for Cry1F protein degradation of Bt corn in soil, which were 0.6 d and 6.9 d, respectively. Herman et al. [45] found that the degradation dynamics of Bt PS149B1 protein conformed neither to exponential nor to biexponential model. Thus, a shift-log model for protein degradation in the soil was proposed. Wang and Feng [46] believed that the shift-log model better represented the degradation dynamics of Cry1Ac protein released from corn straws into the soil. Their studies showed that the exponential model could also reflect the basic features of Cry1Ac protein degradation; however, the fitting precision was poor.

In the present study, the degradation dynamics of Cry1Ac protein was fitted to an exponential model and a shift-log model under different soil water contents and temperatures. The fitting precisions of the exponential models were more accurate (Table 3 and 4). The results showed that the DT_50_ in leaf-soil and bud-soil mixture ranged from 12.29–41.07 d and from 10.17–42.17 d, respectively; while DT_90_ ranged from 41.06–140.75 d and from 33.96–143.90 d, respectively. The estimated results were in consistent with our experimental data. A number of studies have indicated that the DT_50_ of degradation of Cry1Ab protein in soil from Bt corm and Bt rice material could be 2.09–4.08 d [47], 2.2–16 d [26]; while DT_90_ of degradation of it could be 15 d [22], 10.53–38.5 d [47], or 16.1–67.1 d [48]. The DT_50_ and DT_90_ of degradation of Cry1Ac protein determined in the present study were evidently longer than those reported in earlier studies. The different degradation rate for Cry protein in soil may be due to the different crops and the soil matrix as well as environmental conditions. For example, the differences in the C:N ratio between crop species affected decomposition rates and resulted in a two-to three-fold different rate of degradation of residues of Bt cotton, which had a narrower C:N ratio than that of Bt corn [29], [49]. The DT_50_ and DT_90_ of degradation of Cry1Ac protein in leaf-soil mixture and bud-soil mixture had a large difference in the current study, which could be the phenomenon of the composition difference of the leaf and the bud. Previous studies have shown that a large proportion of Cry1Ab protein in Bt corn residues was highly labile and quickly degraded in soil and a small fraction might be protected from decay in relatively recalcitrant residues [35]. The variable degradation rates for Cry protein in soil could also be depended on the differences of soil types and properties. Marchetti et al. [50] found a slower decline of two Cry proteins in a clayey soil than in a sandy soil. The decrease of extractable Cry1Aa protein with incubation time was not related to microbial degradation, but mainly to physicochemical interactions with the surfaces that may decrease immunochemical detectability or enhance protein fixation [20]. Therefore, more efforts are still needed to validate the interactive effects on Cry protein degradation between soil types and properties, and soil environment factors.

## Conclusion

Cry1Ac protein in both the leaves and buds of Bt cotton in soil showed a rapid degradation in the early stage, but a slow decline in the later stage under different soil water content and temperature settings. Our findings suggested that the temperature, as a major factor, determined Cry1AC protein degradation in the soil. The relative higher temperature (25°C, 35°C) was found to be more conducive to degradation of Cry1Ac protein in soil and the greater water content (100% water holding capacity) retarded the process. Moreover, Cry1AC protein degradation dynamics better conformed to an exponential model than to a shift-log model under different soil water contents and temperatures. The greatest Cry1Ac protein degradation rate occurred at 70% water holding capacity and 35°C. The DT_50_ values were 12.29 d and 10.17 d while the DT_90_ values were 41.06 d and 33.96 d in the leaf-soil and bud-soil mixture, respectively. These findings illustrated that under appropriate soil temperature and water content, Cry1Ac protein will not persist and accumulate in soil. The current study also emphasized the significance of interaction effect between soil matrix and the climate factors on Cry1Ac degradation of Bt cotton residues in soil. Additionally, under natural field conditions, the investigation of the effect of environment factor on Cry1Ac protein degradation can be more practical significance. The more field studies are needed to further validate the effect of these factors on the degradation of Cry1Ac protein, which will be useful and urgently necessary for assessing the environment impact of Bt cotton planting on soil ecosystem.
